# Dr. G. G. Crawford’s Pamphlet, and Other Kindred Matter

**Published:** 1871-06

**Authors:** 


### *Dr. G. G. Crawford's Pamphlet, and other Kindred Matters.*

Our attention has been called to a pamphlet extensively circulated, not only among the physicians but the people of this city, and we presume, therefore, the State, purporting to be a statement of Dr. G. G. Crawford, in regard to his connection with the controversy between the late Faculty of the Atlanta Medical College and the Georgia Medical Association, and a reply to a

"Statement of Facts" in the last number of this Journal. While the admission of the author sufficiently establishes all that was asserted in regard to him, we have thought proper to confirm our previous statement by the publication of the testimony of the Professor of Anatomy, under whose immediate supervision he came, while acting as Demonstrator. Dr. O'Keefe states as follows:

DRS. WESTMORELAND—*Gentlemen*:—In accordance with your request I will state that you are entirely correct in the statement that there was great dissatisfaction with Dr. G. G. Crawford upon the part of the class in attendance upon the course 1865 and '66. This dissatisfaction was well known to other members of the Faculty, and was the basis of the action of the Faculty which severed Dr. Crawford's connection with the College.

Very respectfully, etc., D. C. O'KEEFE.

ATLANTA, July 10, 1871.

Another point, and the only one which we regard as calling for any notice from us in the statement of Dr. Crawford, is the attempt to throw dust in the eyes of the profession and the public, and thus to evade the only real issue in this *personal* contest, by introducing the names of other gentlemen into his pamphlet, with the effort to show up their inconsistency, and thus to divert attention from the purpose which has actuated him throughout this contest, as well as to shield himself from the charge of delinquency, which was only presented by us as a last resort, and solely in self-defence, after years of patient waiting, without avail, for the exhaustion of his malice.

We now state once for all, that we (the editors of the ATLANTA MEDICAL AND SURGICAL JOURNAL) are *wholly* and *solely* responsible for everything which has appeared in its editorial columns, and especially for the "Statement of Facts," and we shall not *permit* other members of the late Faculty of the Atlanta Medical College, nor other gentlemen, (and their number is "legion") who, upon investigation, have become convinced of the *purely personal character* of the contest, and now sustain us, to notice any assault arising out of the "Statement of facts connected with the controversy between the late Faculty of the Atlanta Medical College and certain members of the Georgia Medical Association," which we have thought proper to make.

We know nothing of the *private* views formerly entertained by some of these gentlemen, (it is very probable that some of them condemn us, as we now condemn ourselves,) but of one thing we are very sure, and that is, that individuals who are willing so far to violate the proprieties of life, as to resort to the publication of private conversations, taken from their connections, and with a statement of their own inferences, as fact, would not hesitate to pervert or misrepresent, and they will receive as they deserve the contempt of all honorable men. The same remarks are applicable, in a still more decided degree, to those who are endeavoring, by the resurrection of long dead issues, to bring into conflict those who are now friends by a pretended revelation of private, disparaging criticisms, said to have been indulged in a number of years since. For ourselves we should give no credence whatever to the statement of any individual who could resort to such an illegitimate and dishonorable mode of conducting any contest. This is always the verdict of honorable men.

We now dismiss these *personal* matters with the expression of our great gratification (not on account of any triumph which we may have gained) that the medical profession in Georgia, as represented by the Georgia Medical Association and the late Convention at Macon, have put the seal of condemnation upon the further agitation of these disturbing questions, and have given unmistakable indications of their determination to resist any attempt to introduce any one of them into the Association at any future meeting.

For the future we propose to devote this Journal (in connection with others who will probably be connected with its management) exclusively to the interest of the medical profession, and not to allow any matter connected with this or any other personal controversty to soil its pages. We shall probably announce in its next issue the plans now in process for making this a first-class journal in every particular, and one which will, we are sure, be sustained by the contributions and patronage of the best men of the South.

We can assure our friends that the permanency of the JOURNAL is beyond question, and having now, as we hope and believe, disentangled ourselves from the bitter personal controversy which has been forced upon us for the last four years, we now return to the walks of "*Pax et scientia sed veritas sine timore.*"

